# Home practice in Mindfulness-Based Cognitive Therapy and Mindfulness-Based Stress Reduction: A systematic review and meta-analysis of participants' mindfulness practice and its association with outcomes

**DOI:** 10.1016/j.brat.2017.05.004

**Published:** 2017-08

**Authors:** Christine E. Parsons, Catherine Crane, Liam J. Parsons, Lone Overby Fjorback, Willem Kuyken

**Affiliations:** a Interacting Minds Center, Department of Clinical Medicine, Aarhus University, Aarhus, Denmark; b Department of Psychiatry, University of Oxford, Warneford Hospital, Oxford, England, United Kingdom; c Department of Experimental Psychology, University of Bristol, England, United Kingdom; d Danish Center for Mindfulness, Aarhus University, Aarhus, Denmark

**Keywords:** Mindfulness-based cognitive therapy, Mindfulness-based stress reduction, Treatment engagement, Treatment adherence, Homework, Adherence, Mindfulness practice, Meditation practice

## Abstract

Mindfulness-Based Cognitive Therapy (MBCT) and Mindfulness-Based Stress Reduction (MBSR) emphasize the importance of mindfulness practice at home as an integral part of the program. However, the extent to which participants complete their assigned practice is not yet clear, nor is it clear whether this practice is associated with positive outcomes.

For this systematic review and meta-analysis, searches were performed using Scopus and PubMed for studies published through to the end of 2015, reporting on formal home practice of mindfulness by MBSR or MBCT participants.

Across 43 studies (N = 1427), the pooled estimate for participants' home practice was 64% of the assigned amount, equating to about 30 minutes per day, six days per week [95% CI 60–69%]. There was substantial heterogeneity associated with this estimate. Across 28 studies (N = 898), there was a small but significant association between participants’ self-reported home practice and intervention outcomes (r = 0·26, 95% CI 0·19,–0·34).

MBSR and MBCT participants report completing substantial formal mindfulness practice at home over the eight-week intervention, albeit less than assigned amounts. There is a small but significant association between the extent of formal practice and positive intervention outcomes for a wide range of participants.

## Highlights

• Standard mindfulness interventions assign significant home practice.
• This meta-analysis estimated participants' typical practice completion.
• Average mindfulness home practice time equated to 30 min per day, 6 days per week.
• The extent of practice positively correlated with intervention outcomes.

Contemporary psychological treatments require active engagement by participants, both in sessions with a therapist, and in applying and practicing new skills in their lives. Between-session development of these skills through ‘home practice’ is an integral component of treatment. Such home practice is viewed as necessary for participants to gain the insights and skills for the intended treatment outcomes. For cognitive behavioral therapy (CBT), this takes the form of variable assignments, such as self-monitoring, exposure to feared situations, or scheduling of behavioral experiments that extend the therapeutic sessions. Several meta-analyses have provided evidence for a small to moderate association between home assignment completion and CBT treatment outcomes across different psychological disorders (Kazantzis et al., 2010, Mausbach et al., 2010).

Mindfulness-Based Cognitive Therapy (MBCT) and Mindfulness-Based Stress Reduction (MBSR) are manualized, group-based skills training programs that teach mindfulness both in and between sessions. Between-session practice consists of informal and formal home mindfulness practice that trains attention and develops the ability to respond to difficult mental and physical experiences (Kabat Zinn, 1990, Segal et al., 2012). Informal practices encourage mindfulness in everyday life, for example, by deliberately focusing awareness on everyday activities and savouring pleasant experiences. In a formal practice, participants are given guidance as to the nature and content of the practice (e.g., suggestions as to the posture adopted, attitude and how attention is directed).

Typically, participants’ formal practices are supported by audio recordings. In the early weeks of the intervention, participants are gradually introduced to a range of formal meditation practices, focusing initially on mindfulness of the body and the breath, and later the body in movement and mindfulness of thoughts and other mental events. Early practices are intended to support participants in stabilising attention, beginning to notice patterns of mind wandering and increasing the ability to return the mind to an intended focus of attention when mind wandering occurs. Later practices encourage participants to observe patterns of mind wandering in more detail and approach difficult mental content or unpleasant physical sensations with an attitude of curiosity, acceptance and non-judgement. In the final weeks of these interventions, participants are typically encouraged to develop a pattern of formal meditation practice that fits in with their daily life and which will be sustainable beyond the 8-week intervention. In class, teachers review weekly home practice, inviting participants to share their experiences to aid generalisation of learning. These mindfulness home practices are assumed to be critical to therapeutic change. While a growing number of studies have explored the relationship between practice and change, this research is still at an early stage.

This systematic review and meta-analysis aimed to address two key questions about participants' between-session practice in MBSR and MBCT. First, we examined the extent to which participants report completing the assigned formal mindfulness home practice. This is important because psychological ill-health can compromise an individual's capacity to adhere to treatment guidelines (Prince et al., 2007). Furthermore, where interventions involve extensive behavioral components, adherence is often less than ideal (DiMatteo, 2004). Second, we assessed whether there is evidence that completion of formal practice, which is most frequently recorded in MBSR and MBCT studies (Vettese, Toneatto, Stea, Nguyen, & Wang, 2009), is associated with treatment outcomes. It is widely accepted that the full benefit of many effective treatments can only be achieved if the prescribed regime is followed reasonably closely (Osterberg & Blaschke, 2005), but this has not yet been established for mindfulness practice in MBSR and MBCT.

## 1 Methods

This review followed procedures outlined in the Cochrane Handbook for Systematic Reviews (Higgins & Green, 2008) and by the Centre for Reviews and Dissemination (CRD, 2014). The review protocol was registered with PROSPERO [CRD42015029959].

## 2 Search strategy

Searches were performed using Scopus and PubMed for studies published through to the end of 2015, which reported on home practice of mindfulness in MBSR or MBCT. The search terms were: ‘Mindfulness based stress reduction’ or ‘mindfulness based cognitive therapy’, or ‘MBSR’, or ‘MBCT’ and ‘practice’ or ‘homework’ or ‘adherence’ or ‘compliance’ or ‘engagement’ (see Supplementary Materials for search strings). Only primary research presenting novel data on mindfulness practice was included. Two independent reviewers (CP, LP) performed title and abstract screening and full text review using the web-based software platform Covidence, (www.covidence.org; a Cochrane recommended primary screening tool). At full text review, studies were checked to ensure reporting of results from unique, non-overlapping participants. While Covidence does not allow for post hoc calculation of Cohen's Kappa for inter-rater reliability, agreement on screening and data extraction was established between the first two reviewers through discussion for all but 7 issues. These 7 disagreements were resolved with referral to a third reviewer (CC).

We included studies that reported on MBCT or MBSR delivered in line with the format described in the respective manuals, namely an eight-week group program, with class time of 2-2·5 h and one all-day retreat, requiring at-home mindfulness practice for about 45 min, six days per week (Kabat Zinn, 1990, Segal et al., 2013). Studies were excluded if they reported substantial deviations from the standard format such as shortened class times or fewer than eight classes. However, we included studies with reduced home practice requirements (less than the recommended 45 min) as a separate subgroup. Studies were also excluded if they did not report collecting data on participants’ home practice.

We included studies that reported formal home mindfulness practice data (referred to throughout as mindfulness practice) in a format that allowed calculation of average minutes of practice per day, or average number of formal practice sessions per week, for the duration of the course. If studies reported collecting home practice data, but did not report these values, authors were contacted for this information. If authors described the home practice requirements of their intervention, but did not report any actual home practice data, their study was not included in the review. In total, 57 authors were contacted, and 26 responded. Eight authors were able to provide information on formal home practice completion amounts and ten were able to provide information on home practice-outcome associations. Formal practice, defined as the assigned, scheduled home mindfulness sessions (e.g., following a guided meditation, 3-min breathing space), was the focus of our analyses. Formal practice is arguably easier to record in a standard way compared with informal practice, and is more widely reported (Vettese et al., 2009).

In instances of missing or incomplete data, authors were first contacted. Where standard deviations were unavailable after this (12 studies), we compared two methods for SD imputation. The first used an average pooled SD from all other included studies 1 and the second used the largest SD from the available studies. Both yielded similar estimates, so the former method was implemented (Furukawa, Barbui, Cipriani, Brambilla, & Watanabe, 2006). To generate an aggregate estimate of the amount of home practice, a random effects model was implemented with the mean percentage of recommended home practice time (% of 45 min or % of 6 sessions).

## 3 Data extraction and synthesis

Information was extracted from each study as follows (1) the characteristics of the study, where relevant (design, randomization, blinding, therapist qualifications, number of participants, class attendance recorded, type of outcome measures, overall intervention effects), (2) the characteristics of the intervention, including target population (3) the characteristics of participants, including people who did not complete the MBSR/MBCT program (4) home practice details, including recording method, number of participants providing data, amount of formal practice in minutes (or if practice amount was not reported, the number of formal sessions) (M, SD) (5) data on the association between practice (across the entire course) and intervention outcomes. Table 1 presents study characteristics related to the recording of mindfulness practice, class attendance, teacher training and inclusion of a one-day retreat.

**Table 1:** Study characteristics related to recording of practice, class attendance, teacher training and adherence and inclusion of a one-day retreat.

Study	How was practice recorded?	Frequency of practice form collection	Teacher training reported	Did the authors use a scale/measure to check intervention adherence?	Class attendance reported?	All-day retreat
**Studies with standard home practice requirements**						
Baer, Carmody, and Hunsinger (2012)	Weekly logs	Weekly	N	N	Y	All-day retreat
Barnhofer et al. (2009)	Homework records	Not specified	Y	N	Y	Not specified
Blom et al. (2014)	Weekly logs	Weekly	N	N	Y	6 h
Bluth, Gaylord, Nguyen, Bunevicius, and Girdler (2015)	Daily logs	Weekly collection	Y	N	Y	4 h
Britton, Haynes, Fridel, and Bootzin (2010)	Weekly logs	Weekly collection	Y	N	Y	All-day retreat
Britton, Haynes, Fridel, and Bootzin (2012)	Weekly logs	Weekly	Y	N	Y	All-day retreat
Campbell, Labelle, Bacon, Faris, and Carlson (2012)	Daily logs collected at end of course	End of course	Y	N	Y	6 h retreat
Carlson, Speca, Patel, and Goodey (2004)	Daily log	Weekly	N	N	Y	3 h retreat
Carmody and Baer (2008)	Weekly collection	Weekly class	N	N	Y	All-day retreat
Carmody et al. (2008)	Course folder with colour tabs	Weekly class	N	N	N	All-day retreat
Carmody et al. (2011)	Weekly log collected	Weekly	Y	N	Y	All-day retreat
Cole et al. (2015)	Practice logs, frequency not mentioned	Not specified	Y	N	Y	All-day retreat
Collard, Avny, and Boniwell (2008)	Questionnaires at start and end	Not specified	N	N	N	Not specified
Crane et al. (2014)	Daily log	Weekly	Y	Y	Y	Not specified
Day et al. (2014)	Daily online log	Daily	Y	Y	Y	Not specified
Del Re, Flückiger, Goldberg, and Hoyt (2013)	Record after each home practice	End of course	Y	N	Y	All-day retreat
Eisendrath et al. (2015)	Weekly logs	Weekly	Y	N	Y	Not specified
Farb, Segal, and Anderson (2013)	Daily log collected at End of course	End of course	N	N	Y	All-day retreat
Foley, Baillie, Huxter, Price, and Sinclair (2010)	Daily log collected at End of course	End of course	Y	N	Y	All-day retreat
Geschwind (2012)	Not specified	Not specified	Y	N	Y	All-day retreat
Goldsmith et al. (2014)	Weekly logs	Not specified	N	N	N	No retreat
Gross et al. (2011)	Electronic loggers	Daily	Y	N	Y	All-day retreat
Hawley et al. (2014)	Weekly logs	Weekly	Y	Y	N	Not specified
Hoffman et al. (2012)	Weekly sheets	Not specified	Y	N	Y	6 h
Hölzel et al. (2011)	Daily logs	Not specified	N	N	N	6.5 h
Hou et al. (2013)	Weekly collection	Weekly class	Y	N, but sessions videotaped and reviewed	Y	no retreat
Jazaieri et al. (2012)	Weekly phone calls to monitor practice	Weekly	Y	N	N	All-day retreat
Jensen et al., 2012	Daily logs	Not specified	Y	N	Y	7 h retreat
Johansson, Bjuhr, Karlsson, Karlsson, and Rönnbäck (2015)	Daily log	End of course	Y	N	Y	7 h retreat
Kluepfel et al. (2013)	Weekly log	Weekly	Y	Y	Y	All-day retreat
Labelle, Lawlor-Savage, Campbell, Faris, and Carlson (2015)	Weekly logs	Weekly	Y	N	Y	6 h retreat
MacCoon et al. (2012)	Minutes and sessions recorded; frequency not specified	Not specified	Y	N	Y	7 h retreat
Nyklicek and Kuijpers (2008)	Weekly logs	Weekly	N	N	Y	6 h retreat
Ong et al. (2014)	Daily logs	Not specified	Y	N	Y	6 h retreat
Parkin et al. (2014)	Asked weekly about practice time	Weekly	Y	N	N	No retreat
Perich, Manicavasagar, Mitchell, and Ball (2013)	Daily record	Weekly	Y	Y	Y	Not specified
Pickut et al. (2015)	Weekly logs	Weekly class	N	N	Y	no retreat
Pradhan et al. (2007)	Daily logs	Not specified	Y	N	Y	All-day retreat
Ramel, Goldin, Carmona, and McQuaid (2004)	Weekly logs but only half participants filled in; all estimated at follow-up. Data at follow up used	Weekly class	Y	N	N	Not specified
Rimes and Wingrove (2011)	Not specified	Not specified	Y	N	Y	Not specified
Roland et al. (2015)	Daily log	Weekly class	Y	N	Y	3 h retreat
Shallcross et al. (2015)	Not specified	Not specified	Y	Y	Y	Not specified
Shapiro, Brown, and Biegel (2007)	Daily log	Not specified	Y	N	N	Not specified
Shapiro, Jazaieri, and Goldin (2012)	Daily log	Weekly class	Y	N	N	half-day retreat
Shapiro, Oman, Thoresen, Plante, and Flinders (2008)	Daily logs	Not specified	Y	N	Y	No retreat
Vøllestad, Sivertsen, and Nielsen (2011)	Daily log	Not specified	N	N	Y	Half-daYretreat
Whitebird et al. (2013)	Daily log	Not specified	Y	N	Y	5 h retreat
Wong et al. (2011)	Weekly log	Weekly class	Y	N	Y	7 h retreat
Zernicke et al. (2013)	Weekly logs	Weekly class	Y	N	Y	3 h retreat
**Studies with reduced practice requirements**						
Astin (1997)	Daily log	Not specified	N	N	N	no retreat
Bakker et al. (2014)	Calendar diary	Weekly class	Y	N	Y	Not specified
Creswell et al. (2012)	Daily log	Not specified	N	N	Y	7 h retreat
Gross et al. (2011)	Daily log	Regular phone calls	Y	N	Y	Not specified
Hölzel et al. (2011)	Not specified a	Not specified	N	N	Y	All-day retreat
Kimbrough, Magyari, Langenberg, Chesney and Berman (2010)	Daily log	Weekly class	Y	N	Y	5 h retreat
Rosenzweig et al. (2007)	Not specified	Weekly class	N	N	N	7 h retreat
Walach et al. (2007)	Daily log	Not specified	Y	N	N	6 h
Wells et al. (2013)	Not specified	Not specified	Y	N	Y	one day retreat
Wells et al. (2014)	Daily log	Not specified	Y	N	Y	6 h retreat

a Reduced home practice requirements detailed in Hou et al., 2013.

To analyse the association between practice and outcomes, we used the primary outcome at the end of the intervention (around eight weeks), as reported by the study investigators. If this was not specified, we used the most frequently reported measures across studies (BAI, GAD-7, and BDI, PHQ-9, DASS-21 depression subscale, consistent with (Newby, McKinnon, Kuyken, Gilbody, & Dalgleish, 2015). For the two studies reporting longer-term primary outcomes (e.g., hazard of relapse to depression), we obtained outcome measures recorded at the end of the intervention. This decision was made in order to synthesise as much available comparable data as possible. In some instances, authors reported related measures of one physical outcome (e.g., for sleep, sleep initiation, frequency of awakening) and we computed a composite variable (as outlined by Borenstein, Hedges, Higgins, & Rothstein, 2009).

If authors reported standardized regression coefficients, these were used to estimate correlation coefficients (as described by Peterson & Brown, 2005). For two studies (Carmody et al., 2008, Gross et al., 2011), Spearman's rho values were reported and these were converted to Pearson's r (Gilpin, 1993). Where authors reported only that correlations did not reach significance (n = 6), a correlation coefficient was estimated using the study sample size and a conservative p-value of 0·5. Heterogeneity was investigated using forest plots and the *I*^*2*^ statistic.

## 4 Subgroup analyses

To assess the differences between *a priori* identified subgroups of interest (participant group: clinical or nonclinical; intervention primary outcome: physical functioning, psychological functioning or mixed), we conducted subgroup analyses using the mixed effect model approach. We also examined differences across study design (RCTs, non-randomized trials, before and after studies) and differences between MBCT and MBSR.

## 5 Study quality

We examined the risk of bias of included RCTs using the Cochrane ‘Risk of Bias’ tool (Higgins & Green, 2011) and for other study designs, we recorded applicable information. Assessment of study quality was conducted by two independent reviewers (CP, LP) and disagreements were resolved through discussion. Table 2 presents the characteristics related to these quality indices.

**Table 2:** Study characteristics related to quality indices.

Study	Design	Randomized	Randomization procedure	Treatment allocation concealed	Similar at Baseline	Blinded Outcomes	Dropouts recorded	Dropout Reasons	ITT	Power
**Studies with standard home practice requirements**										
Baer et al. (2012)	Before and after	N	N/A	N/A	N/A	N	Y	N	N	N
Barnhofer et al. (2009)	RCT	Y	Y	N	N	Y	Y	N	Y	Y - but underpowered
Blom et al. (2014)	RCT	Y	Y	Y	Y	N	Y	Y	Y	Y
Bluth et al. (2015)	Non-randomized controlled trial	N	N/A	N/A	N	N	Y	Y	N	N
Britton et al. (2010)	RCT	Y	Y	Y	N	Y	Y	N	N	N
Britton et al. (2012)	RCT	Y	Y	N/A	Y	Y	Y	Y	N	N
Campbell et al. (2012)	Non-randomized controlled trial	N	N/A	N/A	Y	N	Y	Y	Y	Y
Carlson et al. (2004)	Before and after	N	N/A	N/A	N/A	N/A	Y	Y	N	N/A
Carmody and Baer (2008)	Before and after	N	N/A	N/A	N/A	N	Y	N	N	N/A
Carmody et al. (2008)	Before and after	N	N/A	N/A	N/A	N	Y	N	N/A	N/A
Carmody et al. (2011)	RCT	Y	Y	N	N	Y	Y	Y	Y	N
Cole et al. (2015)	Before and after	N	N/A	N/A	N/A	N	Y	N/A	N/A	N/A
Collard et al. (2008)	Before and after	N	N/A	N/A	N/A	N/A	Y	N	N/A	N/A
*Crane et al. (2014)	RCT	Y	Y	N	Y	Y	Y	N	N/A	Y
Day et al. (2014)	RCT	Y	N	N/A	Y	N	Y	N	Y	Y
Del Re et al. (2013)	Before and after	N	N/A	N/A	N/A	N	Y	N/A	N	N/A
Eisendrath et al. (2015)	Non-randomized controlled trial	N	N/A	N/A	Y	N	Y	Y	N	N
Farb et al. (2013)	RCT	Y	N	N/A	Y	N	N	N	N	N
Foley et al. (2010)	RCT	Y	Y	N	Y	Y	Y	Y	Y	Y
Geschwind (2012)	RCT	Y	Y	N	Y	N	Y	Y	Y	Y
Goldsmith et al. (2014)	Before and after	N	N/A	N/A	N/A	N/A	Y	N	N/A	N/A
Gross et al. (2011)	RCT	Y	Y	N/A	N	N	Y	N	N	Y
Hawley et al. (2014)	RCT	Y	NA	N	N/A	Y	N	N	N/A	N/A
Hoffman et al. (2012)	RCT	Y	Y	N	Y	Y	Y	Y	Y	Y
Hölzel et al. (2011)	Non-randomized controlled trial	N	N/A	N/A	N/A	N	N	N	N/A	N/A
Hou et al. (2013)	RCT	Y	Y	N	Y	Y	Y	Y	Y	Y
Jazaieri et al. (2012)	RCT	Y	Y	N	Y	N	Y	Y	Y	N
Jensen et al. (2012)	RCT	Y	N	Y	N	Y	Y	Y	N	N
Johansson et al. (2015)	Non-randomized controlled trial	N	N/A	N/A	Y	N	Y	Y	Y	N
Kluepfel et al. (2013)	Before and after	N	N/A	N/A	N/A	N/A	Y	Y	N	N/A
Labelle et al. (2015)	Non-randomized controlled trial	N	N/A	N/A	N/A	N	Y	N	Y	Y
MacCoon et al. (2012)	RCT	Y	Y	Y	Y	Y	Y	Y	Y	Y
Nyklicek and Kuijpers (2008)	RCT	Y	Y	N	Y	Y	Y	N	Y	Y
Ong et al. (2014)	RCT	Y	Y	Y	Y	N	Y	N	Y	Y
Parkin et al. (2014)	Before and after	N	N/A	N/A	N/A	N/A	Y	N	N/A	N/A
Perich et al. (2013)	RCT	Y	N	N	Y	Y	Y	N	Y	Y
Pickut et al. (2015)	RCT	Y	N	N/A	Y	Y	Y	Y	N	N
Pradhan et al. (2007)	RCT	Y	Y	N	N	Y	Y	Y	Y	Y
Ramel et al. (2004)	Non-randomized controlled trial	N	N/A	N/A	Y	N	Y	N	N	N
Rimes and Wingrove (2011)	Before and after	N	N/A	N/A	N/A	Y	N	N/A	N/A	N/A
Roland et al. (2015)	Before and after	N	N/A	N/A	N/A	N	Y	N	N	N/A
Shallcross et al. (2015)	RCT	Y	Y	N/A	Y	Y	Y	N	Y	Y
Shapiro et al. (2007)	Non-randomized controlled trial	N	N/A	N/A	Y	N	Y	N	N	N
Shapiro et al. (2012)	Before and after	N	N/A	N/A	N/A	N/A	Y	N/A	N/A	N
Shapiro et al. (2008)	RCT	Y	Y	N/A	Y	N	Y	N	N	N
Vøllestad et al. (2011)	RCT	Y	N	N/A	Y	N	Y	Y	Y	N
Whitebird et al. (2013)	RCT	Y	Y	N	Y	N	Y	Y	Y	N
Wong et al. (2011)	RCT	Y	Y	Y	Y	Y	Y	N	Y	Y
Zernicke et al. (2013)	RCT	Y	Y	N	Y	N	Y	Y	Y	Y
**Studies with reduced practice requirements**										
Astin (1997)	RCT	Y	N	N	Y	N	Y	N	N	N
Bakker et al. (2014)	RCT	Y	Y	N	Y	N	Y	N	Y	Y
Creswell et al. (2012)	RCT	Y	Y	N	Y	N	Y	N	Y	N
Gross et al. (2004)	pre-post	N	N/A	N/A	N/A	N	Y	N	N/A	Y
Hölzel et al. (2011)	RCT	Y	Y	?	Y	Y	Y	Y	N	N
Kimbrough et al. (2010)	pre-post	N	N/A	N	N/A	N	Y	Y	N/A	Y
Rosenzweig et al. (2007)	pre-post	N	N/A	N/A	N/A	N	Y	Y	N	N/A
Walach et al. (2007)	non randomized	N	N/A	N/A	N/A	N	Y	Y	N	N
Wells et al. (2013)	RCT	Y	Y	N	N	N	N	N	N/A	N
Wells et al. (2014)	RCT	Y	Y	N	N	N	Y	N/A	Y	Y - but underpowered

Note: Randomized = Was the study randomized? PR = Procedure for randomization described? TA = Treatment allocation concealed? Baseline = Similar at baseline? BO = Blind outcome assessments-if unclear note as N, Dropouts = Number of dropouts mentioned. If no mention, score N, DR = Withdrawal reasons stated for dropouts ITT = Intent to treat analysis, Power = Power calculation described. * RCT details described in Williams et al., 2014.

## 6 Study characteristics

**Fig. 1:**
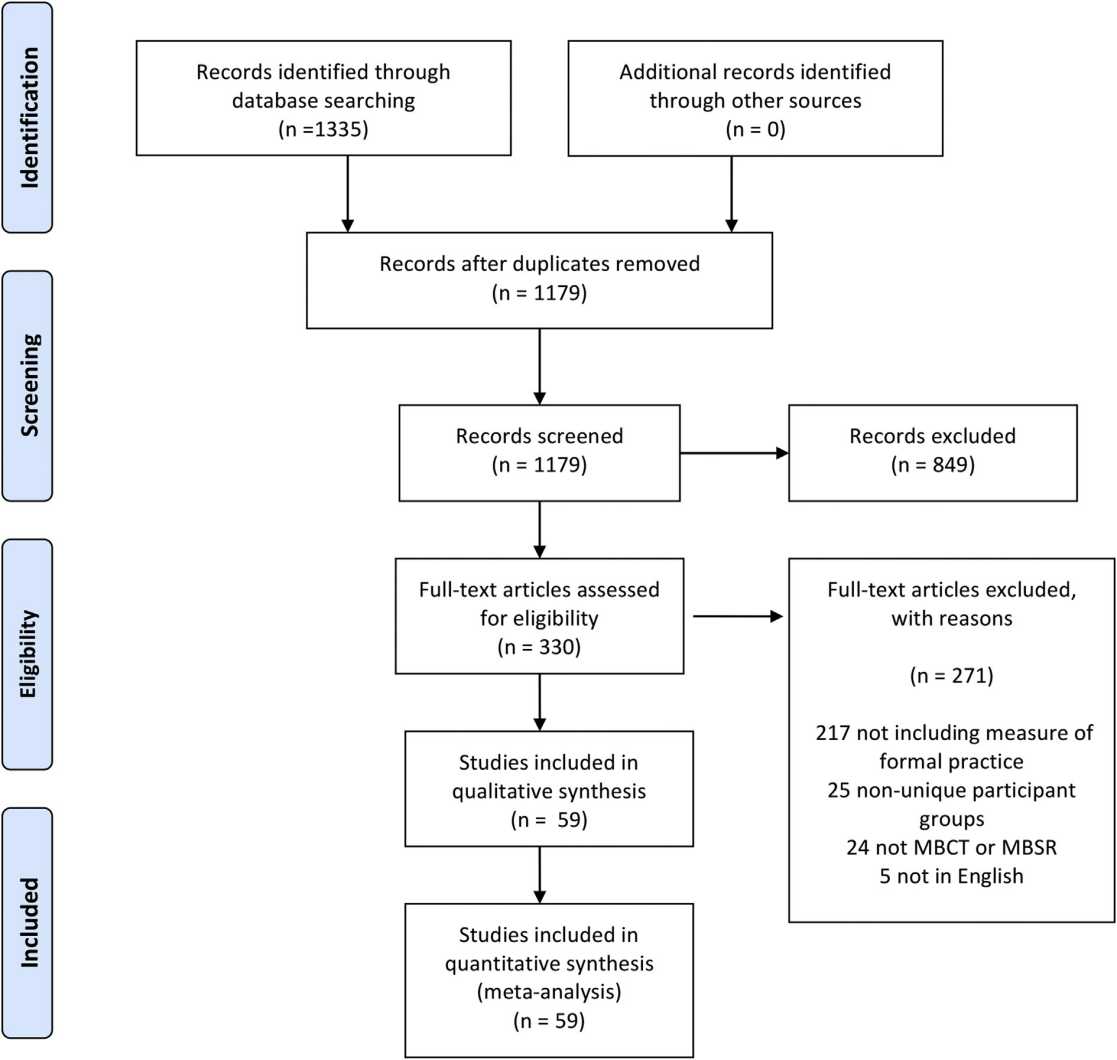
PRISMA diagram of study inclusion.

**Fig. 2:**
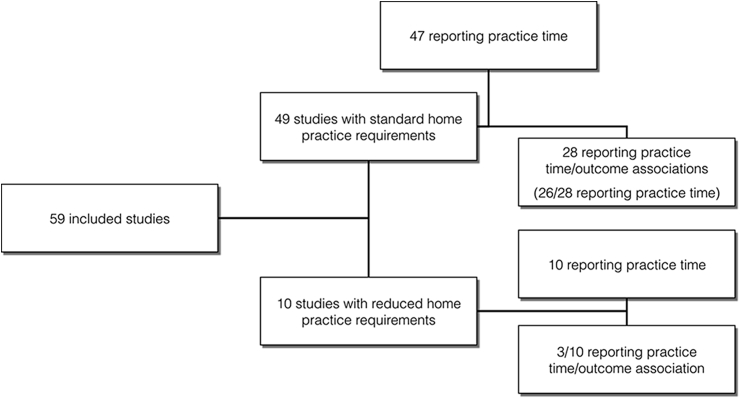
Number of studies reporting on home practice and the association between practice time and outcomes.

Fig. 1 presents the PRISMA flow chart for the included studies. A total of 49 studies were identified that reported mindfulness practice in MBSR/MBCT with standard home practice requirements (45 min). An additional 10 studies were identified that had reduced home practice requirements (N = 141). Two of the 49 studies reported on associations between mindfulness practice and outcomes (Carmody et al., 2008, Eisendrath et al., 2015) but the mean mindfulness practice data were not available. For the ‘standard’ interventions, 28 studies (N = 898) reported associations between practice and intervention outcomes, or provided this data when contacted. For the ‘reduced practice’ interventions, three studies reported information on the correlation between practice and outcomes, or provided this data when contacted (see Fig. 2).

Of the 49 studies included with standard home practice requirements, 28 were RCTs, 15 were uncontrolled ‘before and after’ studies and six were non-randomized controlled trials. The majority of studies reported on clinical populations (clinical n = 41; nonclinical = 8). Most reported on MBSR groups (n = 34), while 12 reported on MBCT, and a further two reported on mixed MBCT and MBSR groups. There were a variety of populations treated in these studies, but the most common were depression/anxiety (n = 10), cancer (n = 5) and insomnia (n = 4). The majority examined primary outcomes related to psychological functioning (e.g., depression, anxiety, stress; n = 33), but a substantial proportion examined physical functioning (e.g., blood pressure, sleep, BMI; n = 12), and a smaller number reported ‘mixed’ outcomes (e.g., quality of life; n = 4). Of the 10 studies included with reduced home practice requirements, 6 were RCTs, 3 were uncontrolled ‘before and after’ studies and one was a non-randomized controlled trial. All of these studies examined MBSR participants (see Table 1).

In general, studies reported on the training of the intervention teacher (36/49 of standard format studies, 6/10 reduced practice format) and number of classes attended by participants (37/49 of standard format studies, 7/10 reduced practice format). Only a small number of studies reported using a scale to assess intervention adherence by the teacher (5/49 standard format studies).

## 7 Risk of bias within randomized controlled trials

The methodological quality of the studies reporting RCTs varied widely (see Table 2). Twenty-six (76%) reported adequate generation of random sequencing, 6 (17%) reported adequately concealing group allocation, and 18 (51%) reported appropriate blinding of outcome assessments. Dropouts were reported for 31 studies (86%), but only a minority reported dropout reasons (46%). Twenty-two studies (63%) reported intent to treat analysis. Seventeen studies reported power calculations, but two of these reported that the sample size was underpowered.

## 8 How much practice do participants complete in standard format MBSR/MBCT?

Mindfulness practice was typically recorded in paper diaries and collected during the weekly classes (Table 1). Practice records were described as logs, diaries, calendars, or forms (e.g., “Tick boxes were used by participants to record each element of home practice alongside a space to make any free response comments on their home practice for their own benefit and that of the class instructor” from Crane et al., 2014.). One study used an online daily diary recording method (Day et al., 2014), one used electronic loggers (Gross et al., 2011) and one used weekly phone calls to monitor practice (Jazaieri, Goldin, Werner, Ziv, & Gross, 2012). Most studies reported participants’ practice as an average amount (minutes, hours) per week or per day, allowing calculation of an overall percentage of recommended practice completion. Four studies reported only the frequency of practice per week (see Table 1), and this was expressed as a percentage of the recommended 6 times per week.

Four studies were identified as extreme outliers. Three studies reported that participants completed more than 45 min (>100%) of practice and it was not possible to establish if this was combined reports of formal and informal home practice for two cases (Cole et al., 2015, Pradhan et al., 2007). One study was an outlier in the other direction (Del Re et al., 2013), with participants reporting an average of 14% of home practice (SD = 3·14%). These studies were excluded from pooled estimates because of the uncertainty involved in these participant reports, but their exclusion did not impact on the pattern of results.

**Fig. 3:**
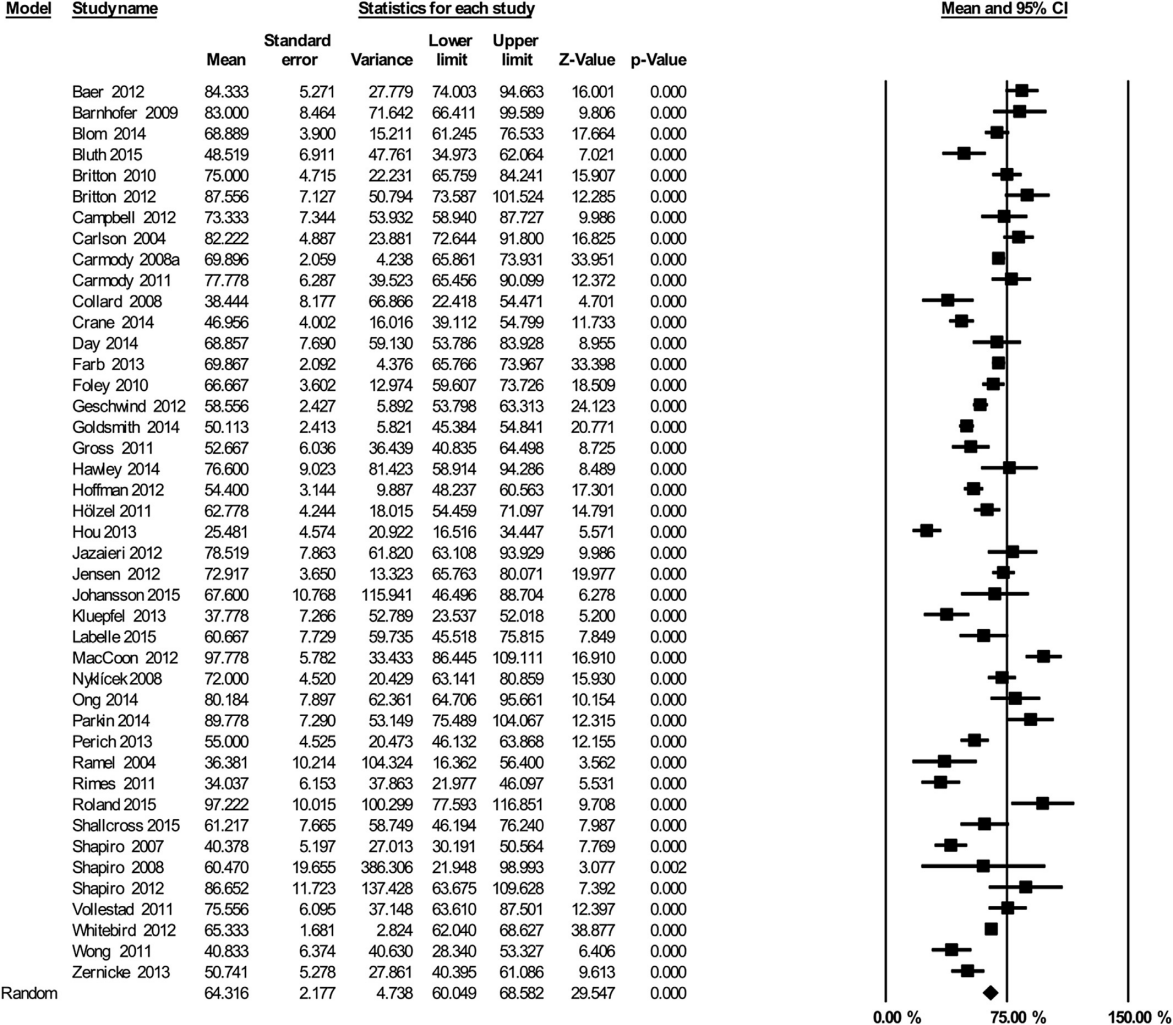
Mindfulness practice in standard format MBSR/MBCT: Mean percentage of recommended practice (45 min, six days per week) reported across 43 studies.

Across the 43 included studies, the pooled estimate for participants’ practice was 64% of the recommended amount (which equates to approximately 29 min per day, Fig. 3, 95% CI 60–69%]. However, there was substantial heterogeneity associated with this estimate, as reflected by *I*^2^ = 89%.

There were no significant differences in practice for studies examining clinical populations (n = 35, 65·95%, 95% CI 62·06 - 69·83%) and nonclinical populations (n = 8; 56·32%, 95% CI 36·67–76·33; Q = 0·84, df = 1, p = 0·36). We compared studies where primary intervention outcomes related to physical functioning, psychological functioning or a ‘mixed’ outcome (e.g., quality of life). Tau squared values were pooled across subgroups because of the limited number of ‘mixed’ outcome studies as recommended by Borenstein et al., (2009). There were no significant differences in home practice between studies examining primary intervention outcomes related to psychological functioning (n = 29; 67·93%, 95% CI 59·9–75·95%), physical functioning (n = 12, 67·95; 95% CI 59·83–76·07%), and mixed outcomes (n = 3, 66·7%; 95% CI 49·87–83·53%; Q = 1·35, df = 2, p = 0·51).

Comparing MBCT and MBSR, we found no significant differences in home practice reports (MBCT: n = 11, 61.08%, 95% CI 52·7–69·44%; MBSR: n = 21, 64·4%; 95% CI 59.26–69.52%; Q = 0.44, df = 1, p = 0·51).

Finally, three comparisons were performed related to practice recording, study design and study quality. First, studies were compared that asked participants to fill in ‘daily’ logs of practice (n = 18) compared with ‘weekly’ logs (n = 18). While heterogeneity was lower across studies reporting the use of daily logs *(*I^*2*^ = 84%) compared to weekly logs (I^2^ = 93%), there was no evidence for significant differences in home practice across these two groups (daily: 64·6% 95% CI 59–70·3%; weekly: 65·6%, 95% CI 56·9–74·25%, Q = 0·03, df = 1, p = 0·86). Second, there was also no evidence for significant differences in home practice by study design, comparing RCTs (n = 26; 64·9%, 95% CI 59·4–70·47%), non-randomized trials (n = 7; 61·5%, 95% CI 49·1–73·9%) and before and after studies (n = 10; 64·3%, 95% CI 55·25–73·3%; Q = 0·25, df = 2, p = 0·88). Third, we also restricted analysis to RCTs with a low risk of bias, as indicated by three criteria (see Table 2 reporting of randomization procedure, blinding of outcomes, recording of attrition). Heterogeneity remained high in this subgroup of RCTs (n = 13, 66%, 95% CI 55·76–77·1%, I^2^ = 93%). Finally, we performed a sensitivity analysis excluding studies with small sample sizes (less than 20, n = 14). The pooled estimate of participants' practice was 62·7% (CI 57·79–67·75%), but again heterogeneity was substantial (I^2^ = 90%).

## 9 Studies with reduced home practice requirements

**Fig. 4:**
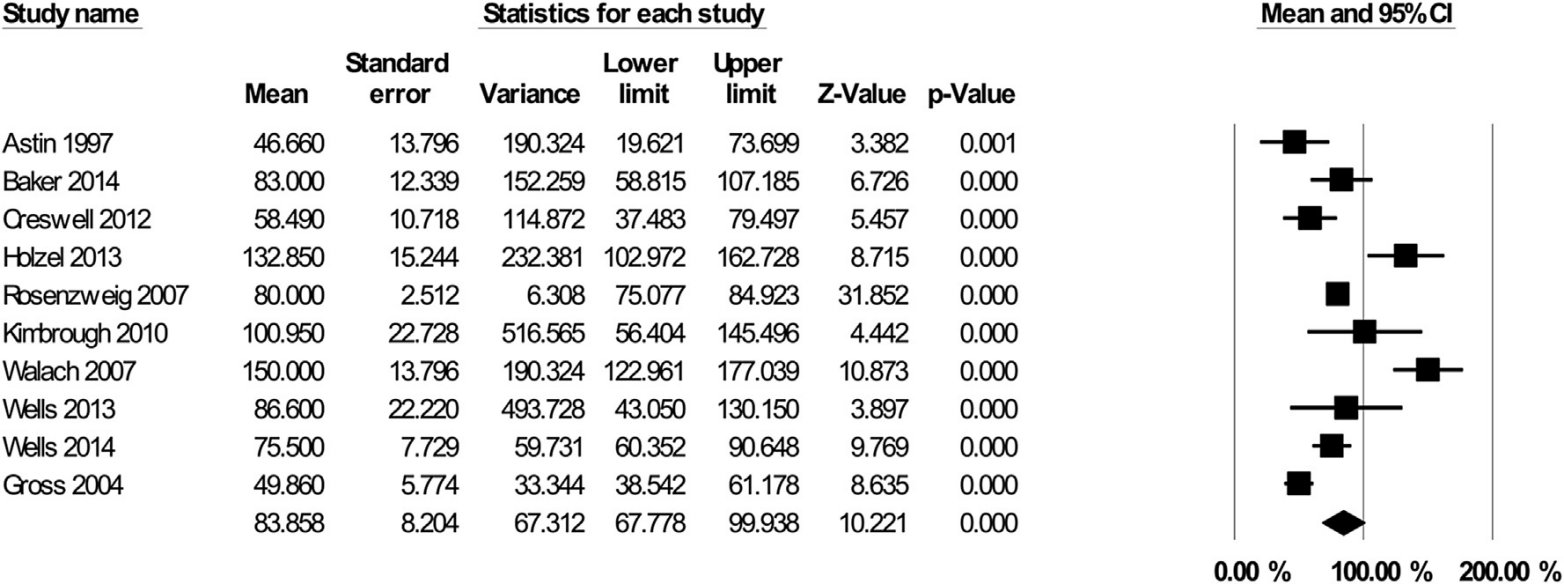
Mindfulness practice in MBSR/MBCT with reduced home practice requirements: Mean percentage completed of requested practice reported across 10 studies.

In 10 studies (N = 141), participants were asked to practice for less than the standard amount (i.e., less than 45 min per day/6 days per week or 270 min). We calculated the amount of practice participants reported completing as a percentage of the amount requested. On average, these studies asked participants to practice for 180 min (SD = 43) across the week (e.g., 30 min per day, 6 days per week, or 45 min per day, 5 days per week). The pooled estimate for participants' practice was 83.86% of the requested amount (which equates to approximately 151 min per week, 95% CI 67.78–99.94%, see Fig. 4). However, there was substantial heterogeneity associated with this estimate, as reflected by *I*^2^ = 87.89%. We expressed the practice time of these intervention participants as a percentage of the standard practice time, to compare interventions with different practice requirements. A subgroup comparison showed that participants in the ‘standard practice’ interventions reported completing more practice than those in the ‘reduced practice’ interventions (n = 10, 52.24%, 95% CI 43.18–61.3%, Q = 5.6, df = 1, p = 0·02).

## 10 Publication bias

We found evidence suggesting a publication bias for the studies reporting on quantity of home practice (see Appendix 2, Fig. 1). Duval and Tweedie's Trim and Fill method, testing for missing studies to the left side of the mean effect based on a random effects model, suggested five missing studies, with an imputed point estimate of 61% (95% CI 56·8 - 65·6%). However, given the substantial heterogeneity associated with the home practice pooled estimate, the Trim and Fill imputed estimate should also be interpreted cautiously (Terrin, Schmid, Lau, & Olkin, 2003).

## 11 Is there an association between home practice and intervention outcomes?

**Fig. 5:**
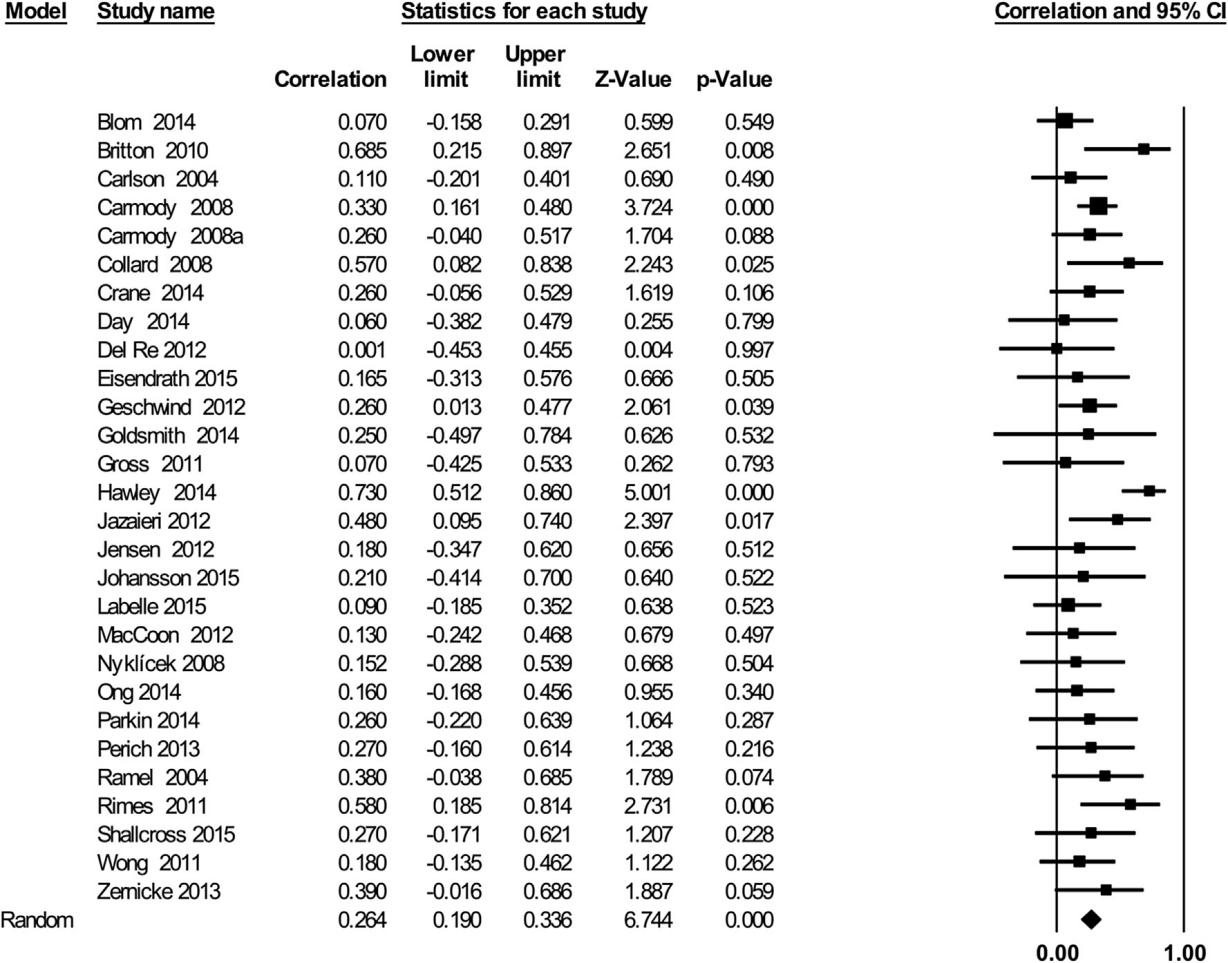
The association between home practice and intervention outcomes across 28 studies.

Across the 28 studies, there was a small but significant association between participants’ home practice and intervention outcomes (Fig. 5, r = 0·26, 95% CI 0·19,–0·34, Z = 6·74, p < 0.0001). Heterogeneity of effects between studies did not appear to be substantial (*I*^*2*^ = 17.43%; p = 0·21).

Subgroup analyses showed no evidence of a difference in the relationship between home practice and outcomes across clinical (n = 22, r = 0·25, 95% CI 0·17–0·34) and nonclinical populations (n = 5, r = 0·29, 95% CI 0·07–0·48, Z = 2·5, Q = ·09, df = 1, p = 0·76). There was also no evidence of an overall significant difference in the home practice/outcome association across studies grouped by intervention outcome type (Q = 5·52, df = 2, p = 0·17). However, the majority of available studies reported on primary psychological outcomes (n = 19, r = 0·3, 95% CI 0·21 -·38, Z = 6·37, p < 0·0001), while a smaller number reported on physical outcomes (n = 6, r = 0·, 95% CI -0·001–0·31, Z = 1·95, p = 0·05). Only two studies reported on the association between SHFP and ‘mixed’ (quality of life) intervention outcomes and neither study reported a significant association between practice and outcome (r = 0·13, 95% CI 0·17–0·411, Z = ·85, p = 0·39). There was also no evidence of an effect of study design, comparing RCTs (n = 15, r = ·26, 95% CI 0·143–0·376, Z = 4·21, p < 0·0001), non-randomized trials (n = 4, r = ·19, 95% CI -0·04–0·4, Z = 1·6, p = 0·1), and before and after studies (n = 8, r = ·29, 95% CI 1·48–0·42, Z = 3·9, p < 0·0001; Q = 0·61, df = 2, p = 0·74).

Restricting analysis to RCTs only (n = 15), the association between home practice and outcomes remained small, but significant (r = 0·26, 95% CI 0·143–0·376, Z = 4·21, p < 0·0001). Finally, a meta-regression was performed to examine whether the strength of the association between home practice and outcomes differed dependent on the mean amount of home practice. There was no evidence for a linear impact of mean home practice on the practice-outcome association (Beta = −0·0004, SE = ·003, Z = 0·16, p = 0·86). We found no evidence for a publication bias for studies examining home practice and outcome associations (Supplementary Materials, Fig. 2).

Finally, for the 10 studies with reduced home practice requirements, two reported that there was no significant association between practice and outcomes, but did not report statistical details (Astin, 1997, n = 12; Kimbrough et al., 2010; n = 23). Another study found no significant relationships between home practice completion and post treatment changes in self-reported loneliness (Creswell et al., 2012, *r* (13) = 0.35, p = 0.2^2^). The remaining 7 studies did not report on the association between home practice and outcomes.

## 12 Discussion

The available evidence suggests that in standard format MBCT and MBSR, participants complete about 60% of assigned formal home practice, where it is recorded and reported. This equates to around 30 min per day, six days a week and represents a substantial time commitment, albeit less than suggested in standard intervention formats (Kabat Zinn, 1990, Segal et al., 2013). We found no evidence for significant differences in practice completion across clinical and nonclinical participant groups nor between studies targeting psychological and physical health outcomes. We found evidence for a small, significant association between practice and outcomes across the 28 standard format studies. This significant association held across clinical and nonclinical participant groups and across physical and psychological treatment outcomes.

However, there was substantial heterogeneity associated with the pooled estimates of participants' home practice. Even within *a priori* subgroups, heterogeneity was high and we could not readily identify its source using indices of study quality or study design. Within individual studies, participants’ practice reports were variable, as were reports across studies. This suggests a need for a greater understanding of the individual-level factors affecting reports of mindfulness home practice, as well as study-level factors. We also examined the small body of studies with reduced home practice requirements, which comprised MBSR interventions only. We found that participants in these studies practiced significantly less overall than those asked to practice for the standard amount of time (i.e., 151 min vs. 174 mins per week).

Participants’ perception of treatment plausibility and expectancy of positive outcome have been shown to have a small but significant impact on treatment outcomes for psychological therapies more generally (meta-analysis; Constantino, Arnkoff, Glass, Ametrano, & Smith, 2011). However, such effects have not been clearly established for MBCT or MBSR participants (Crane et al., 2014, but see also; Snippe et al., 2015). Participant personality traits such as compliance or conscientiousness may also be important. Other factors implicated in CBT homework completion, namely motivation to change (Helbig & Fehm, 2004), teacher competence and reviewing of home assignments (Weck, Richtberg, Esch, Höfling, & Stangier, 2013) might be investigated in MBCT/MBSR. Study level factors including therapist adherence to MBSR or MBCT protocols, or indeed an interaction between these factors, might also be relevant. We could not examine these factors because of infrequent investigation.

### 12.1 The association between home practice and outcomes

We found a small to moderate association between participants' home practice and treatment outcome, where participants are asked to practice for the standard amount of time. There was no evidence of heterogeneity of effects. The strength of the association was similar to that reported in meta-analyses of CBT homework assignments and outcomes (Kazantzis et al., 2010, Mausbach et al., 2010). This finding suggests that there is value in supporting and encouraging participants’ home practice in MBCT and MBSR. Mindfulness practice is often conceptualized as a form of mental training (Tang, Hölzel, & Posner, 2015) and like physical training, greater practice may confer greater benefit. Given the small size of the practice and outcome association, exploration of additional participant engagement variables, such as class attendance, alongside home practice, may be fruitful. Finally, there was insufficient data from studies with reduced home practice requirements to address the practice/outcome question.

These findings should also be considered in relation to the small number of dismantling trials that have tested whether mindfulness is the “active ingredient” in MBSR (MacCoon et al., 2012) and MBCT (Williams et al., 2014). For MBSR, a trial with a nonclinical participant sample suggests that it is no more effective than an active control condition (health enhancement programme) in improving well-being indices. For MBCT, evidence suggests that it is more beneficial for patients with recurrent depression at increased vulnerability (history of childhood trauma) than an active control condition. However, there was no significant advantage for MBCT over the active treatment in the overall patient sample. Overall, these trials raise questions about the active components of treatment, but they do not directly test the importance of home practice itself in MBSR or MBCT.

### 12.2 Limitations

While our findings suggest that home practice is clinically important, there are a number of caveats. We found some evidence of underreporting of participants' home practice, with lower practice amounts less likely to be reported. However, we did not find any evidence of a publication bias for studies reporting on the association between practice and outcomes. Nonetheless, it should be noted that the majority of studies of MBSR/MBCT do not report on participants' home practice. Furthermore, the home practice reports examined here were from participants' who had completed the 8-week interventions. This review draws attention to the need to record and report where possible, home practice from all participants (completers and non-completers). This would provide a broader understanding of participants’ behavior outside of class time and its impact on outcomes.

The quality of the evidence included here is another limitation. We did not restrict inclusion based on study design because the aim was to examine home practice completion rather than the efficacy of MBSR or MBCT as interventions. Nevertheless, many of the included RCTs were at risk for bias from lack of outcome assessment blinding, allocation concealment, high attrition and lack of intention-to-treat analysis. These sources of bias have been identified in previous systematic reviews of RCTs of meditation interventions generally (e.g., Goyal et al., 2014).

There is much potential for improved methodology in studying home practice and outcomes in MBSR and MBCT. Current estimates of mindfulness practice rely on participants’ retrospective self-reports, but it is unclear how this relates to their actual practice behavior. Related to this, included studies typically reported asking participants to complete daily diaries or weekly forms. We found no differences between mindfulness practice recorded using either form type. However, it is difficult to ascertain the actual frequency with which participants completed these forms.

Furthermore, few studies provided details on the specific forms filled in by participants (e.g., form by Crane et al., 2014). Development and widespread use of standard home practice reporting forms would be helpful in ensuring consistency in participant experience and in reporting across studies. Future use of smartphone apps, text message reminders to fill in practice diaries, or online portals, may support participants in recording home practice. In addition, this would provide researchers with a means to assess the frequency and timing of practice recording. Smartphone apps may be particularly valuable as a method of recording informal practices in real-time (e.g., when participant undertake unscheduled ‘additional breathing spaces’ in response to stressful events, and ‘noticings’ – bringing mindful awareness to moments in daily life). Future studies may also examine whether specific practices (e.g., body scan, yoga) are more robustly correlated with treatment outcomes than others.

Furthermore, participants' practice ‘quality’ may be crucial (Del Re et al., 2013), but again this presents an inherent measurement challenge. We also did not examine informal practice, which has been investigated in two recent studies but was not found to affect intervention outcomes (Crane et al., 2014, Hawley et al., 2014). However, as has been widely discussed, informal practice is more challenging to quantify when compared with formal practice, which has a more standard duration with audio guidance. Teacher competence in reviewing home practice, and providing formative feedback, may be particularly important in obtaining insights into practice behavior, in increasing engagement with practice, and indeed in increasing the beneficial effects of practice on outcome.

A further limitation of the current evidence is that studies investigating the formal home practice and outcome association are correlational. An arguably better strategy to investigate whether home practice is necessary for positive treatment outcomes might involve randomly assigning participants to ‘MBSR/MBCT as usual’ compared with a ‘no formal practice’ format. Finally, we chose to focus our analysis on studies reporting outcomes immediately post intervention and the majority of MBCT/MBSR studies to date have been over a relatively short time frame. This allowed us to synthesize a reasonably large body of studies. However, MBCT has been shown to protect against relapse to depression (Kuyken et al., 2015), an outcome that requires longer assessment periods. An important future avenue therefore will be to examine practice, and its continuation beyond the eight-week intervention, and longer-term effects.

## Author contributions

All authors contributed to the study design and writing of the manuscript. CP and LP performed the literature searches and data extraction. CP performed the data analysis and CP and CC performed the data interpretation.

## Footnotes

1 The average SD from the included studies was 31.7%.

2 Data provided by authors when contacted.
